# An Improved Marriage in Honey Bees Optimization Algorithm for Single Objective Unconstrained Optimization

**DOI:** 10.1155/2013/370172

**Published:** 2013-07-14

**Authors:** Yuksel Celik, Erkan Ulker

**Affiliations:** ^1^Department of Computer Programming, Karamanoglu Mehmetbey University, Karaman, Turkey; ^2^Computer Engineering Department, Selcuk University, Konya, Turkey

## Abstract

Marriage in honey bees optimization (MBO) is a metaheuristic optimization algorithm developed by inspiration of the mating and fertilization process of honey bees and is a kind of swarm intelligence optimizations. In this study we propose improved marriage in honey bees optimization (IMBO) by adding Levy flight algorithm for queen mating flight and neighboring for worker drone improving. The IMBO algorithm's performance and its success are tested on the well-known six unconstrained test functions and compared with other metaheuristic optimization algorithms.

## 1. Introduction

Optimization means to find the best among the possible designs of a system. In other words, for the purpose of minimizing or maximizing a real function, selecting real or integer values from an identified range, placing these into the function and systematically examining or solving the problem is referred to as optimization. For the solution of optimization problems, mathematical and heuristic optimization techniques are used. In problems with wide and large solution space, heuristic algorithms heuristically produce the closest results to the solution, without scanning the whole solution space and within very short durations. Metaheuristic algorithms are quite effective in solving global optimization problems [1]. The main metaheuristic algorithms are genetic algorithm (GA) [2], simulated annealing (SA) [3], particle swarm optimization (PSO) [4], ant colony optimization (ACO) [5], differential evolution (DE) [6], marriage in honey bees optimization (MBO) [7, 8], artificial bee colony algorithm (ABC) [9] and evolutionary algorithms (EAs) [9, 10].

Performance of algorithms carrying out nature-inspired or evolutionary calculations can be monitored with their application on the test functions of such algorithms. Karaboga and Basturk implemented the artificial bee colony (ABC) algorithm, which they proposed from the inspiration of the food searching activities of honey bees, on unconstrained test functions [11]. Digalakis and Margaritis developed two algorithms titled as the generational replacement model (GRM) and the steady state replacement model by making modifications on the genetic algorithm and monitored their performances on unconstrained test functions [12]. By combining the GA and SA algorithms, Hassan et al. proposed the geno-simulated annealing (GSA) algorithm and implemented it on the most commonly used unconstrained test functions [13]. In order to obtain a better performance in the multidimensional search space, Chatterjee et al. suggested the nonlinear variation of the known PSO, the non-PSO algorithm, and measured its performance on several unconstrained test functions [14]. By integrating the opposition-based learning (OBL) approach for population initialization and generation jumping in the DE algorithm, Rahnamayan et al. proposed the opposition-based DE (ODE) algorithm and compared the results they obtained from implementing the algorithm on the known unconstrained test functions with DE [15]. It is difficult to exhibit a good performance on all test functions. Rather than expecting the developed algorithm to provide accurate results on all types of problems, it is more reasonable to determine the types of problems where the algorithm functions well and decide on the algorithm to be used on a specific problem. 

Test functions determine whether the algorithm will be caught to the local minimum and whether it has a wide search function in the search space during the solution.

In 2001, Abbass [8] proposed the MBO algorithm, which is a swarm intelligence based and metaheuristic algorithm predicated on the marriage and fertilization of honey bees. Later on, Abbass and Teo used the annealing approach in the MBO algorithm for determining the gene pool of male bees [16]. Chang made modifications on MBO for solving combinatorial problems and implemented this to the solution. Again, for the solution of infinite horizon-discounted cost stochastic dynamic programming problems, he implemented MBO on the solution by adapting his algorithm he titled as “Honey Bees Policy Iteration” (HBPI) [17]. In 2007, Afshar et al. proposed MBO algorithm as honey bee mating optimization (HBMO) algorithm and implemented it on water resources management applications [18]. Marinakis et al. implemented HBMO algorithm by obtaining HBMOTSP in order to solve Euclidan travelling salesman problem (TSP) [19]. Chiu and Kuo [20] proposed a clustering method which integrates particle swarm optimization with honey bee mating optimization. Simulations for three benchmark test functions (MSE, intra-cluster distance, and intercluster distance) are performed.

In the original MBO algorithm, annealing algorithm is used during the queen bee's mating flight, mating with drones, generation of new genotype, and adding these into the spermatheca. In the present study, we used Levy flight [1] instead of the annealing algorithm. Also, during the improvement of the genotype of worker bees, we applied single neighborhood and single inheritance from the queen. We tested the IMBO algorithm we developed on the most commonly known six unconstrained numeric test functions, and we compared the results with the PSO and DE [21] algorithms from the literature.

This paper is organized as follows: in Section 2, the MBO algorithm and unconstrained test problems are described in detail.  Section 3 presents the proposed unconstrained test problems solution procedure using IMBO.  Section 4 compares the empirical studies and unconstrained test results of IMBO, MBO, and other optimization algorithms.  Section 5 is the conclusion of the paper.

## 2. Material and Method

### 2.1. The Marriage in Honey Bee Optimization (MBO) Algorithm

#### 2.1.1. Honey Bee Colony

Bees take the first place among the insects that can be characterized as swarm and that possess swarm intelligence. A typical bee colony is composed of 3 types of bees. These are the queen, drone (male bee), and workers (female worker). The queen's life is a couple of years old, and she is the mother of the colony. She is the only bee capable of laying eggs. 

Drones are produced from unfertilized eggs and are the fathers of the colony. Their numbers are around a couple of hundreds. Worker bees are produced from fertilized eggs, and all procedures such as feeding the colony and the queen, maintaining broods, building combs, and searching and collecting food are made by these bees. Their numbers are around 10–60 thousand [22]. 

Mating flight happens only once during the life of the queen bee. Mating starts with the dance of the queen. Drones follow and mate with the queen during the flight. Mating of a drone with the queen depends of the queen's speed and their fitness. Sperms of the drone are stored in the spermatheca of the queen. The gene pool of future generations is created here. The queen lays approximately two thousand fertilized eggs a day (two hundred thousand a year). After her spermatheca is discharged, she lays unfertilized eggs [23].

#### 2.1.2. Honey Bee Optimization Algorithm

Mating flight can be explained as the queen's acceptance of some of the drones she meets in a solution space, mating and the improvement of the broods generated from these. The queen has a certain amount of energy at the start of the flight and turns back to the nest when her energy falls to minimum or when her spermatheca is full. After going back to the nest, broods are generated and these are improved by the worker bees crossover and mutation.

Mating of the drone with the queen bee takes place according to the probability of the following annealing function [8]:

(1) $$\begin{array}{c} \text{prob}f\left(Q,D\right)=e^{-\text{difference}/\text{speed}}, \end{array}$$

where (*Q*, *D*) is the probability of the drone to be added to the spermatheca of the *Q* queen (probability of the drone and queen to mate) and Δ(*f*) is the absolute difference between *D*'s fitness and *Q*'s fitness. *f*(*Q*) and *S*(*t*) are the speed of the queen at *t* time. This part is as the annealing function. In cases where at first the queen's speed is high or the fitness of the drone is as good as the queen's fitness, mating probability is high. Formulations of the time-dependent speed *S*(*t*) and energy *E*(*t*) of the queen in each pass within the search space are as follows:

(2) $$\begin{array}{c} S\left(t+1\right)=\alpha \times S\left(t\right),\\ E\left(t+1\right)=E\left(t\right)-\gamma . \end{array}$$

Here, *α* is the factor of ∈[0, 1] and *γ* is the amount of energy reduction in each pass. On the basis of (1) and (2), the original MBO algorithm was proposed by Abbas [8] as shown in Algorithm 1.

### 2.2. Unconstrained Numerical Benchmark Functions

Performance of evolutionary calculating algorithms can be monitored by implementing the algorithm on test functions. A well-defined problem set is useful for measuring the performances of optimization algorithms. By their structures, test functions are divided into two groups as constrained and unconstrained test functions. Unconstrained test functions can be classified as unimodal and multimodal. While unimodal functions have a single optimum within the search space, multimodal functions have more than one optimum. If the function is predicated on a continuous mathematical objective function within the defined search space, then it is a continuous benchmark function. However, if the bit strings are not defined and continuous, then the function is described as a discreet benchmark function [24]. Alcayde et al. [25] approach a novel extension of the well-known Pareto archived evolution strategy (PAES) which combines simulated annealing and tabu search. They applied this several mathematical problems show that this hybridization allows an improvement in the quality of the nondominated solutions in comparison with PAES Some of the most commonly known test functions are as follows. We have solved well-known six unconstrained single objective numeric benchmark function. The details of the benchmark functions are given in Table 1.

## 3. IMBO for Unconstrained Test Functions

In the original MBO mating possibility of the queen bee in the mating flight is calculated through the annealing function. In the proposed study Improved MBO (IMBO) algorithm was obtained by improving the MBO algorithm through the replacement of the annealing algorithm with the Levy flight algorithm in order to enable the queen to make a better search in the search space. Flight behaviors of many animals and insects exhibited the typical characteristics of Levy flight [26]. In addition, there are many studies to which Levy flight was successfully adapted. Pavlyukevich solved a problem of nonconvex stochastic optimization with the help of simulated annealing of Levy flights of a variable stability index [27]. In biological phenomena, Viswanathan et al. used Levy flight in the search of biologic organisms for target organisms [28]. Reynolds conducted a study by integrating Levy flight algorithm with the honey bees' strategies of searching food [29]. Tran et al. proposed Levy flight optimization (LFO) for global optimization problems, implemented it on the test functions, and compared the results they obtained with simulated annealing (SA) [30]. By adapting Levy flight algorithm instead of the gaussian random walk in the group search optimizer (GSO) algorithm developed for Artificial neural network (ANN), Shan applied the algorithm on a set of 5 optimization benchmark functions [31].

In general terms, Levy flight is a random walk. The steps in this random walk are obtained from Levy distribution [1]. Levy flight is implemented in 2 steps. While the first is a random selection of direction, the second is the selection of a step suitable for Levy distribution. While direction has to be selected from a homogenous distribution region, step selection is a harder process. Although there are several methods for step selection, the most effective and simplistic one is the Mantegna algorithm. 

Mantegna algorithm is calculated as shown in the following equation:

(3) $$\begin{array}{c} s=\frac{u}{{\left|v\right|}^{1/\beta }}. \end{array}$$

Here, the *v* is obtained by taking the magnitude of the genotype as basis. 

*u* on the other hand is calculated as shown in the following equation;

(4) $$\begin{array}{c} \sigma_{u}={\left\{\frac{\Gamma \left(1+\beta \right)\sin(\pi \beta /2)}{\Gamma \left[\left(1+\beta \right)/2\right]\beta 2^{(\beta -1)/2}}\right\}}^{1/\beta },\;\sigma_{u}=1.\;\; \end{array}$$

While in this equation *β* is 0 ≤ *β* ≤ 2, is the Γ is the Gamma function, and calculated as follows:

(5) $$\begin{array}{c} \Gamma \left(z\right)=\int_{0}^{\infty }t^{z-1}e^{-t}dt. \end{array}$$

In consequence, the direction of the next step is determined with the *u* and *v* parameters, and step length is found by placing *u* and *v* into their place in the Mantegna algorithm (3). Based on *S*, new genotype is generated as much as random genotype size, and the generated genotype is added to the previous step. Consider

(6) $$\begin{array}{c} s=\propto_{0}\left(x_{j}^{\left(t\right)}-x_{i}^{\left(t\right)}\right)⊕\text{L}\overset{´}{\text{e}}\text{vy}\left(\beta \right)\sim 0.01\frac{u}{{\left|v\right|}^{1/\beta }}\left(x_{j}^{\left(t\right)}-x_{i}^{\left(t\right)}\right). \end{array}$$

Creation of the new genotype of this step is completed by subjecting the new solution set obtained, that is, the genotype to the maximum and minimum controls defined for the test problem and adjusting deviations if any. Accordingly, through these implemented equations, the queen bee moves from the previous position to the next position, or towards the direction obtained from Levy distribution and by the step length obtained from Mantegna algorithm as follows:

(7) $$\begin{array}{c} L_{ij}=x_{ij}+s*\text{rand}\left(\text{size}\left(x_{ij}\right)\right). \end{array}$$

In the crossover operator, the genotype of the queen bee and all genotypes in the current population are crossed over. Crossover was carried out by randomly calculating the number of elements subjected to crossover within Hamming distance on the genotypes to be crossed over.

In the improvement of the genotype (broods) by the worker bees single neighborhood and single inheritance from the queen was used. Consider

(8) $$\begin{array}{c} W_{ij}=x_{ij}+\left(x_{ij}-x_{kj}\right)\emptyset_{ij}, \end{array}$$

where *∅*_*ij*_ is a random (0, 1) value, *x*_*i*_ is the current brood genotype, *x*_*k*_ is the queen genotype, *j* is a random value number of genotype. In this way, and it was observed that the developed IMBO algorithm exhibits better performance than the other metaheuristic optimization algorithms.

The MBO algorithm we modified is shown in Algorithm 2.

## 4. Experimental Results

**Figure 1:**
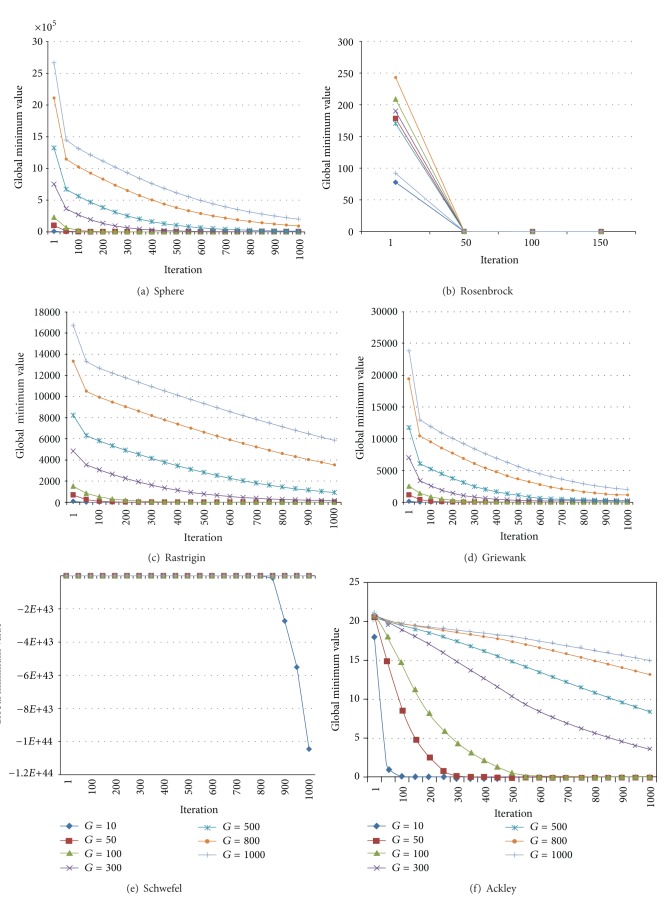
Mean global minimum convergence graphs of benchmark functions in 1000 generations for genotype sizes (*G*) of 10, 50, 100, 300, 500, 800 and 1000.

In this study, we used Sphere, Rosenbrock, Rastrigin, Griewank, Schwefel, and Ackley unconstrained test problems; a program in the MatLab 2009 programming language was developed for the tests of MBO and IMBO. Genotype sizes of 10, 50, 100, 300, 500, 800, and 1000 were taken for each test. Population size (Spermatheca Size) was accepted as *M* = 100. At the end of each generation, mean values and standard deviations were calculated for test functions. Each genotype was developed for 10,000 generations. Each improvement was run for 30 times. Global minimum variance graphs of each test function for IMBO are presented in Figure 1.

Examining the six test functions presented in Figure 1 shows that, at first, global optimum values were far from the solution value in direct proportion to genotype size. Accordingly, for large genotypes, or in other words in cases where the number of entry parameters is high, convergence of the proposed algorithm to the optimum solution takes a longer time. The test results for MBO and IMBO are given in Tables 2 and 3. 

When Tables 2 and 3 were examined, it is seen that genotype size increases in all functions of MBO IMBO algorithms and the global minimum values get away from the optimal minimum values. In Table 2, the MBO algorithm Sphere function, 10, 50, 100 the size of genotype reached an optimum value while the other genotype sizes converge to the optimal solution. It was observed that Rosenbrock function minimum is reached optimal sizes in all genotypes. It was observed that rastrigin function 10, 50, 100, Griewank function 50, 100 genotype sizes in the optimal solution was reached. It was observed that except Schwefel function, other function and genotype sizes, the optimal solution was reached close to values.

When Table 3 was examined, it was seen that, while the size of genotype increased, IMBO algorithm Sphere, Rastrigin, Griewank, Schwefel, and Ackley function, were getting away from the optimal minimum. It was seen that Sphere function, 10, 50, 100 the size of genotype reached an optimum value while the other genotype sizes converges to the optimal solution. It was observed that, except for the size of 10 genotypes Rosenbrock function, but all other genotypes sizes optimal minimum was reached. Rastrigin function 10, 50, Griewank function 50, 100 to the optimal solution was observed to have reached the optimal solution. The other functions and sizes of genotype were observed to have reached the values close to the optimal solution.

Comparative results of the best and mean solutions of the MBO and IMBO algorithms are presented in Table 4.

According to Table 4, it is seen that, when compared with the MBO algorithm and IMBO algorithm according to genotype, IMBO exhibited better performance than MBO in all genotypes sizes. When it was thought that total better or equal cases were represented with “+” mark, MBO algorithm, a total of 19 “+” available, and IMBO algorithm, a total of 36 pieces of the “+” were available. Accordingly, IMBO's MBO algorithm demonstrates a better performance.

CPU time results of the all genotype sizes for MBO are given in Table 5 and for IMBO are given in Table 6.

In Tables 5 and 6, it was seen that, when CPU time values were analyzed, depending upon the size in the same proportion as genotype problem, solving time took a long time. Again in these tables, when solution CPU time of MBO and IMBO algorithm was analyzed, IMBO algorithm solves problems with less CPU time than MBO algorithm.

For 10, 50, 100, and 1000 problem sizes of unconstrained numeric six benchmark functions, comparisons were made between test results of IMBO algorithm and the algorithms in literature, including DE, PSO, ABC [32], bee swarm optimization, (BSO) [33], bee and foraging algorithm (BFA) [34], teaching-learning-based optimization (TLBO) [35], bumble bees mating optimization (BBMO) [36] and honey bees mating optimization algorithm (HBMO) [36].  Table 7 presents the comparison between experimental test results obtained for 10-sized genotype (problem) on unconstrained test functions of IMBO algorithm and the results for the same problem size in literature including PSO, DE, ABC, BFA and BSO optimization algorithms; while the comparison of success of each algorithm and IMBO algorithm is given in Table 8.  Table 9 shows the comparison between experimental test results obtained for 50-sized genotype (problem) on unconstrained test functions of IMBO algorithm and the results for the same problem size in literature including TLBO, HBMO and BBMO optimization algorithms, while the comparison of success of each algorithm and IMBO algorithm is given in Table 10.  Table 11 shows the comparison between experimental test results for 100-sized genotype (problem) on unconstrained test functions of IMBO algorithm and the results for the same problem size in literature including PSO, DE, and ABC optimization algorithms, while the comparison of success of each algorithm and IMBO algorithm is given in Table 12.  Table 13 shows the comparison between experimental test results obtained for 1000-sized genotype (problem) on unconstrained test functions of IMBO algorithm and the results for the same problem size in the literature including PSO, DE, and ABC optimization algorithms, while the comparison of success of each algorithm and IMBO algorithm is given in Table 14.

Tables 7, 11, and 13 demonstrate that, as the problem size increases in ABC, DE, and PSO, the solution becomes more distant and difficult to reach. However, the results obtained with IMBO showed that, despite the increasing problem size, optimum value could be obtained or converged very closely. There are big differences among the results obtained for 10, 100, and 1000 genotype sizes in DE and PSO; however, this difference is smaller in IMBO algorithm, which indicates that IMBO performs better even in large problem sizes. In Tables 7 and 8, it is seen that IMBO performs equally to DE and ABC and better than PSO, BFA and BSO. In Tables 9 and 10 showing the comparison of IMBO with LBO, HBMO, and BBMO for genotype (problem) size 50, it is seen that IMBO performs better than all the other algorithms. In Tables 11 and 12 showing the comparison of IMBO with DE, PSO and ABC algorithms on problem size 100, it is seen that IMBO performs equally to ABC and better than DE and PSE. In Tables 13 and 14 showing the comparison of IMBO with DE, PSO and ABC on problem size 1000, IMBO is seen to perform better than all the other algorithms. 

## 5. Conclusion

In the proposed study, we developed a new IMBO by replacing annealing algorithm in the queen bee's mating flight with the Levy flight algorithm and using single inheritance and single neighborhood in the genotype improvement stage. We tested the MBO algorithm we improved on the most commonly known six unconstrained numeric benchmark functions. We compared the results obtained with the results of other metaheuristic optimization algorithms in the literature for the same test functions. 

In order to observe the improvement of IMBO, the experimental test results of MBO and IMBO were compared for 10, 50, 100, 300, 500, 800, and 1000 problem sizes. Consequently, IMBO algorithm was concluded to perform better than MBO algorithm. Furthermore, according to CPU time of problem solving process, IMBO algorithm works in shorter CPU times. The test results obtained with IMBO were compared with the results of DE, ABC, PSO, BSO, BFA; TLBO, BBMO and HBMO in the literature. 

Accordingly, IMBO is observed to perform equally to or a little better than other algorithms in comparison with small genotype size, while IMBO performs much better than other algorithms with large genotype size. A total of 14 comparisons were made between experimental results of IMBO and other optimization algorithms in literature, and it showed better performances in 11 comparisons, and equal performances in 3 comparisons. 

In future studies, different improvements can be made on the MBO algorithm and tests can be made on different test functions. Also, comparisons can be made with other metaheuristic optimization algorithms not used in the present study.

## Figures and Tables

**Algorithm 1:**
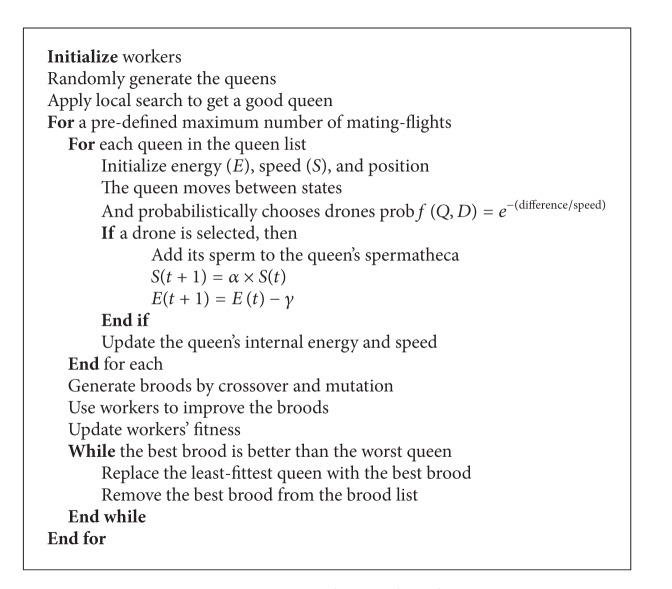
Original MBO algorithm [8].

**Algorithm 2:**
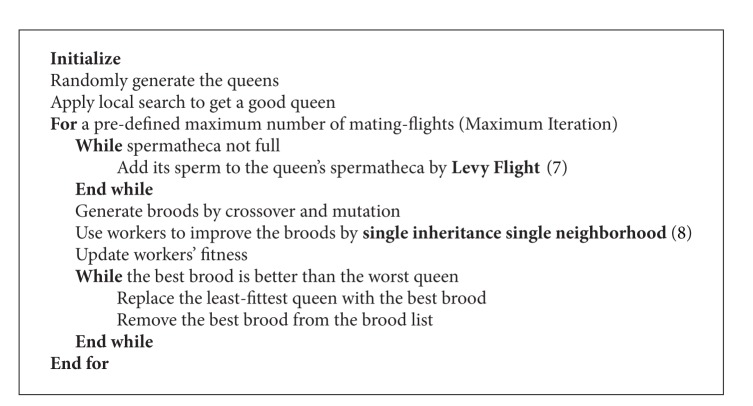
Proposed IMBO algorithm.

**Table 1:** Unconstrained test functions.

No.	Function name	Formula	Range	Optimum *f*(*x*)
1	Sphere	$f\left(x\right)=\overset{n}{\underset{i=1}{\sum }}x^{2}$	[5.12, 5.12]^*n*^	0
2	Rosenbrock	$f\overset{\to }{\left(x\right)}=\overset{n-1}{\underset{i=1}{\sum }}100{\left(x_{i+1}-x^{2}\right)}^{2}+{\left(x_{i}-1\right)}^{2}$	[−2.04, 2.048]^*n*^	0
3	Rastrigin	$f\overset{\to }{\left(x\right)}=10\cdot n+\overset{n}{\underset{i=1}{\sum }}\left({x_{i}^{2}}_{}-10\cdot \cos\left(2\pi x_{i}\right)\right)$	[−5.12, 5.12]^*n*^	0
4	Griewank	$f\overset{\to }{\left(x\right)}=\overset{n}{\underset{i=1}{\sum }}\frac{x_{i}^{2}}{4000}-\prod \cos\left(\frac{x_{i}}{\sqrt{i}}\right)+1$	[−600, 600]^*n*^	0
5	Schwefel	$f\overset{\to }{\left(x\right)}=418.9829\cdot n+\overset{n}{\underset{i=1}{\sum }}x_{i}\cdot \text{sin⁡}\left(\sqrt{\left|x_{i}\right|}\right)$	[−500, 500]^*n*^	−418.9829∗*n*
6	Ackley	$f\overset{\to }{\left(x\right)}=20+e-20\cdot e^{-0.2\cdot \sqrt{(1/n)\sum_{i=1}^{n}x_{i}^{2}}}-e^{(1/n)\sum_{i=1}^{n}\text{cos⁡}(2\pi x_{i})}$	[−32.768, 32.768]^*n*^	0

**Table 2:** Test results of the MBO algorithm for the genotype sizes of 10, 50, 100, 300, 500, 800, and 1000; number of runs = 30; SD: standard deviation, AV: global minimum average; generation = 10000.

Genotype size		10	50	100	300	500	800	1000
Sphere	AV	0	0	0	1.97*E* − 26	1.21*E* − 13	2.06*E* − 06	8.26*E* − 04
	SD	9.81*E* − 01	3.36*E* + 01	1.41*E* + 02	1.19*E* + 03	2.66*E* + 03	6.82*E* + 03	1.84*E* + 04
Rosenbrock	AV	0	0	0	0	0	0	0
	SD	4.73*E* − 03	8.67*E* − 03	8.29*E* − 03	1.55*E* − 02	7.28*E* − 03	8.09*E* − 03	0
Rastrigin	AV	0	0	0	6.0633*E* − 13	7.88048*E* − 12	3.33194*E* − 05	0.002606
	SD	0	0	0	3.95279*E* − 13	1.80594*E* − 12	3.09109*E* − 05	0.002843
Griewank	AV	0.010377126	0	0	3.40468*E* − 16	7.26498*E* − 08	1.1388*E* − 06	0.000141
	SD	0.026549182	0.32563139	1.09610828	8.02049039	70.78602045	36.67034057	108.177558
Schwefel	AV	−9.99*E* + 223	−2.96*E* + 211	−1.00*E* + 183	−2.37*E* + 306	−1.02*E* + 305	−1.10*E* + 305	−1.10*E* + 305
	SD	3.81*E* + 22	1.94*E* + 16	5.13*E* + 26	4.31*E* + 35	7.31*E* + 35	3.39*E* + 32	7.89*E* + 35
Ackley	AV	4.08562*E* − 15	8.23045*E* − 15	7.3922*E* − 09	8.49609*E* − 14	9.13234*E* − 10	0.000227233	0.000452387
	SD	0.006649746	0.01783530	0.61180347	0.05005151	0.507695929	1.288113922	0.287375496

**Table 3:** Test results of the IMBO algorithm for the genotype sizes of 10, 50, 100, 300, 500, 800, and 1000; number of runs = 30; SD: standard deviation, AV: global minimum average; generation = 10000.

Genotype size		10	50	100	300	500	800	1000
Sphere	AV	0	0	0	1.38*E* − 28	1.47*E* − 13	2.78*E* − 07	0.0001224
	SD	0	0	0	8.97*E* − 29	1.90*E* − 15	8.50*E* − 08	3.09*E* − 05
Rosenbrock	AV	2.25*E* − 07	0	0	0	0	0	0
	SD	2.24*E* − 06	0	0.1425029	0	0	0.2440021	0
Rastrigin	AV	0	0	4.54*E* − 15	5.68*E* − 13	3.60*E* − 12	6.13*E* − 07	0.0002386
	SD	0	0	3.18*E* − 14	4.78*E* − 13	1.25*E* − 12	3.14*E* − 07	9.13*E* − 05
Griewank	AV	0.0002219	0	0	3.48*E* − 16	9.15*E* − 13	1.78*E* − 07	3.26*E* − 05
	SD	0.0012617	0	0	3.85*E* − 17	1.88*E* − 14	5.43*E* − 08	6.73*E* − 06
Schwefel	AV	−1.21*E* + 23	−1.08*E* + 25	−6.17*E* + 29	−1.21*E* + 40	−1.76*E* + 36	−2.96*E* + 39	−1.64*E* + 39
	SD	7.28*E* + 44	1.06*E* + 26	3.52*E* + 30	6.52*E* + 40	5.78*E* + 36	2.14*E* + 40	1.49*E* + 40
Ackley	AV	3.73*E* − 15	8.13*E* − 15	1.57*E* − 14	7.49*E* − 14	1.76*E* − 05	3.30*E* − 05	0.0006221
	SD	1.42*E* − 15	9.94*E* − 16	2.30*E* − 15	5.11*E* − 15	3.09*E* − 05	9.57*E* − 06	0.0001513

**Table 4:** Success rates of MBO when compared with those of the IMBO algorithm. + indicates that the algorithm is better while − indicates that it is worse than the other. If both algorithms show similar performance, they are both +.

Genotype size	10		50		100		300		500		800		1000	
Function	MBO	IMBO	MBO	IMBO	MBO	IMBO	MBO	IMBO	MBO	IMBO	MBO	IMBO	MBO	IMBO
Sphere	+	**+**	+	+	+	+	−	+	+	−	−	+	−	+
Rosenbrock	+	**−**	+	+	+	+	+	+	+	+	+	+	+	+
Rastrigin	+	**+**	+	+	+	−	−	+	−	+	−	+	−	+
Griewank	−	**+**	+	+	+	+	+	−	−	+	−	+	−	+
Schwefel	−	**+**	−	+	−	+	−	+	−	+	−	+	−	+
Ackley	−	**+**	−	+	−	+	−	+	+	−	−	+	+	−

Total	3	5	4	6	4	5	2	5	3	4	1	6	2	5

**Table 5:** CPU time results of the MBO algorithm for the genotype sizes of 10, 50, 100, 300, 500, 800, and 1000; number of runs = 1; iteration = 10000.

Genotype size	Sphere	Rosenbrock	Rastrigin	Griewank	Schwefel	Ackley
10	02:45:095	02:36:827	03:12.552	05:00.894	03:25.141	03:20.882
50	05:39:832	05:35.729	06:15.572	08:22.590	07:30.842	06:20.845
100	15:47:269	17:13.912	17:36.329	18:34.517	17:29.746	15:55:318
300	1:09:07:083	1:07:07.602	1:12:21.617	1:16:02.748	1:17:11.700	1:16:04.994
500	2:03:31:669	2:03:54.118	2:08:22.112	2:03:11.670	2:06:25.689	2:09:27.164
800	3:23:52:834	2:51:14.834	2:53:25.547	3:00:11.898	3:06:41.792	2:52:34.737
1000	4:31:56.752	3:35:18.224	3:40:03.753	3:45:45.894	3:54:20.884	3:38:12.446

**Table 6:** CPU time results of the IMBO algorithm for the genotype sizes of 10, 50, 100, 300, 500, 800, and 1000; number of runs = 1; iteration = 10000.

Genotype size	Sphere	Rosenbrock	Rastrigin	Griewank	Schwefel	Ackley
10	35.303	31.949	40.716	01:08.110	46.831	42.245
50	43.134	39.749	49.592	01:20.714	01:21.869	48.141
100	54.475	50.466	01:00.980	01:36.439	01:57.109	58.983
300	1:42.213	01:39.778	01:52.445	02:45.033	04:41.472	01:41.151
500	2:32.662	02:11.836	02:31.180	03:26.841	07:30.062	02:26.812
800	3:49.415	03:16.452	03:49.633	05:00.754	11:15.094	03:49.227
1000	4:48.789	4:02:082	04:37.822	06:05.650	14:15.259	04:42.845

**Table 7:** The mean solutions obtained by the PSO, DE, ABC, BFA, BSO, and IMBO algorithms for 6 test functions over 30 independent runs and total success numbers of algorithms. Genotype size: 10; (—): not available value, SD: standard deviation, AV: global minimum average.

Problem		PSO	DE	ABC	IMBO	BFA	BSO
Sphere	ORT	4.13*E* − 17	4.41*E* − 17	4.88*E* − 17	0	0.000031	8.475*E* − 123
	SD	7.71*E* − 18	8.09*E* − 18	5.21*E* − 18	0	0.00024	3.953*E* − 122
Rosenbrock	ORT	0.425645397	4.22*E* − 17	0.013107593	2.25*E* − 07	7.2084513	3.617*E* − 7
	SD	1.187984439	1.09*E* − 17	0.008658828	2.24*E* − 06	9.436551	5.081*E* − 5
Rastrigin	ORT	7.362692992	0.099495906	4.76*E* − 17	0	0.003821	4.171*E* − 64
	SD	2.485404145	0.298487717	4.40*E* − 18	0	0.006513	7.834*E* − 64
Griewank	ORT	0.059270504	0.008127282	5.10*E* − 19	0.000221881	3.209850	3.823*E* − 46
	SD	0.03371245	0.009476456	1.93*E* − 19	0.00126167	4.298031	6.679*E* − 46
Schwefel	ORT	−2654.033431	−4166.141206	−4189.828873	−1.21*E* + 23	—	—
	SD	246.5263242	47.37533385	9.09*E* − 13	7.289*E* + 44	—	—
Ackley	ORT	4.67*E* − 17	4.86*E* − 17	1.71*E* − 16	3.73*E* − 15	0.000085	7.105*E* − 19
	SD	8.06*E* − 18	6.55*E* − 18	3.57*E* − 17	1.42*E* − 15	0.000237	5.482*E* − 18

**Table 8:** Comparative results of IMBO with PSO, DE, ABC, BFA, and BSO algorithms over 30 independent runs for genotype size 50. + indicates that the algorithm is better while − indicates that it is worse than the other, (—): not available value. If both algorithms show similar performance, they are both +.

Problem	IMBO-PSO		IMBO-DE		IMBO-ABC		IMBO-BFA		IMBO-BSO	
	IMBO	PSO	IMBO	DE	IMBO	ABC	IMBO	BFA	IMBO	BSO
Sphere	+	−	+	−	+	−	+	−	+	−
Rosenbrock	+	−	−	+	+	−	+	−	+	−
Rastrigin	+	−	+	−	+	−	+	−	+	−
Griewank	+	−	+	−	−	+	+	−	−	+
Schwefel	−	+	−	+	−	+	—	—	—	—
Ackley	−	+	−	+	−	+	+	−	−	+

Total	4	2	3	3	3	3	5	0	3	2

**Table 9:** The mean solutions obtained by the TLBO, HBMO, BBMO, and IMBO algorithms for 6 test functions over 30 independent runs and total success numbers of algorithms. Genotype size: 50; (—): not available value, SD: standard deviation, AV: global minimum average.

Problem		IMBO	TLBO	HBMO	BBMO
Sphere	AV	0	0.00	0.67	0.00
	SD	0	0.00	—	—
Rosenbrock	AV	0	47.0162	46.07	24.37
	SD	0.142502861	3.56*E* − 01	—	—
Rastrigin	AV	4.54747*E* − 15	2.03*E* − 12	4.03	1.59*E* − 08
	SD	3.18323*E* − 14	5.46*E* − 12	—	—
Griewank	AV	0	0.00	1.44*E* − 02	0.00
	SD	0	0.00	—	—
Schwefel	AV	−6.17561*E* + 29	−20437.84	—	—
	SD	3.52272*E* + 30	1.48*E* + 02	—	—
Ackley	AV	1.57*E* − 14	3.55*E* − 15	—	—
	SD	2.30*E* − 15	8.32*E* − 31	—	—

**Table 10:** Comparative results of IMBO with TLBO, HBMO, and BBMO algorithms over 30 independent runs for genotype size 50. + indicates that the algorithm is better while − indicates that it is worse than the other, (—): not available value. If both algorithms show similar performance, they are both +.

Problem	IMBO-TLBO		IMBO-HBMO		IMBO-BBMO	
	IMBO	TLBO	IMBO	HBMO	IMBO	BBMO
Sphere	+	−	+	−	+	+
Rosenbrock	+	−	+	−	+	−
Rastrigin	+	−	+	−	+	−
Griewank	+	+	+	−	+	+
Schwefel	−	+	—	—	—	—
Ackley	−	+	—	—	—	—

Total	4	3	4	0	4	2

**Table 11:** The mean solutions obtained by the DE, PSO, ABC, and IMBO algorithms for 6 test functions over 30 independent runs and total success numbers of algorithms. Genotype size: 100; (—): not available value, SD: standard deviation, AV: global minimum average.

Problem		PSO	DE	ABC	IMBO
Sphere	AV	5.14*E* − 16	8.84*E* − 17	1.08*E* − 15	0
	SD	3.12*E* − 16	4.29*E* − 17	1.04*E* − 16	0
Rosenbrock	AV	113.143751	132.3488752	0.054865327	0
	SD	48.99432331	41.72265261	0.045566135	0.142502861
Rastrigin	AV	148.2486456	133.1138439	1.08*E* − 15	4.54747*E* − 15
	SD	17.76489083	106.6728854	8.99*E* − 17	3.18323*E* − 14
Griewank	AV	0.048643996	0.000739604	4.92*E* − 17	0
	SD	0.063166266	0.002218812	4.25*E* − 18	0
Schwefel	AV	−20100.36156	−31182.49983	−41898.28873	−6.17561*E* + 29
	SD	1763.156655	2078.47339	3.30*E* − 10	3.52272*E* + 30
Ackley	AV	0.732022399	2.14*E* − 16	4.21*E* − 15	1.57*E* − 14
	SD	0.755456829	4.53*E* − 17	3.09*E* − 16	2.30*E* − 15

**Table 12:** Comparative results of IMBO with DE, PSO, and ABC algorithms over 30 independent runs for genotype size 100. + indicates that the algorithm is better while − indicates that it is worse than the other. If both algorithms show similar performance, they are both +.

Problem	IMBO-PSO		IMBO-DE		IMBO-ABC	
	IMBO	PSO	IMBO	DE	IMBO	ABC
Sphere	+	−	+	−	+	
Rosenbrock	+	−	+	−	+	
Rastrigin	+	−	+	−	−	+
Griewank	+	−	+	−	+	−
Schwefel	−	+	−	+	−	+
Ackley	+	−	+	−	−	+

Total	5	1	5	1	3	3

**Table 13:** The mean solutions obtained by the DE, PSO, ABC, and IMBO algorithms for 6 test functions over 30 independent runs and total success numbers of algorithms. Genotype size: 1000; (—): not available value, SD: standard deviation, AV: global minimum average.

Problem		PSO	DE	ABC	IMBO
Sphere	AV	9723.034942	329214.6744	0.058275686	0.000122371
	SD	3920.944041	917847.3604	0.021093306	3.09627*E* − 05
Rosenbrock	AV	1679629.019	14373397912	2603.968539	0
	SD	648462.4744	361340776.6	599.4022496	0
Rastrigin	AV	2722.799729	1674.782779	735.8480014	0.000238574
	SD	83.14754621	96.86409615	24.75231998	9.13969*E* − 05
Griewank	AV	86.03568115	266.1639753	0.10290266	3.2663*E* − 05
	SD	29.1502045	335.3504904	0.068217103	6.73212*E* − 06
Schwefel	AV	−187704.1438	−252854.5198	−350890.8062	−1.64729*E* + 39
	SD	11097.95553	17724.02042	2279.801625	1.49786*E* + 40
Ackley	AV	8.741445965	17.47129372	3.200412604	0.000622099
	SD	0.784830594	3.815946124	0.133628837	0.000151332

**Table 14:** Comparative results of IMBO with DE, PSO, and ABC algorithms over 30 independent runs for genotype size 1000. + indicates that the algorithm is better while − indicates that it is worse than the other. If both algorithms show similar performance, they are both +.

Problem	IMBO-PSO		IMBO-DE		IMBO-ABC	
	IMBO	PSO	IMBO	DE	IMBO	ABC
Sphere	+	−	+	−	+	−
Rosenbrock	+	−	+	−	+	−
Rastrigin	+	−	+	−	+	−
Griewank	+	−	+	−	+	−
Schwefel	−	+	−	+	−	+
Ackley	+	−	+	−	+	−

Total	5	1	5	1	5	1
